# Marginal adaptation and fracture resistance of virgilite-based occlusal veneers with varying thickness

**DOI:** 10.1186/s12903-024-04071-6

**Published:** 2024-03-05

**Authors:** Amr Rizk, Jylan El-Guindy, Ahmed Abdou, Reem Ashraf, Citra Kusumasari, Farid Emad Eldin

**Affiliations:** 1 Fixed Prosthodontics division, Department of Prosthetic Dentistry, Faculty of Dentistry, King Salman International University, South Sinai, Egypt; 2 Fixed Prosthodontics department, Faculty of Dentistry, Cairo University, Cairo, Egypt; 3 Faculty of Dentistry, Al-Ayen University, Thi‑Qar, Iraq; 4 Biomaterials Division, Department of Prosthetic Dentistry, Faculty of Dentistry, King Salman International University, South Sinai, Egypt; 5 Conservative Dentistry department, Faculty of Dentistry, Universitas Indonesia, Jakarta, Indonesia; 6 Fixed Prosthodontics department, Faculty of Dentistry, British University in Egypt, Cairo, Egypt

**Keywords:** Vertical marginal gap, Tabletop, Advanced lithium disilicate, Virgilite, Composite CAD/CAM blocks, Thermodynamic aging, Fracture resistance, Weibull analysis

## Abstract

**Statement of problem:**

CAD/CAM occlusal veneers have been developed for minimally invasive prosthetic restoration of eroded teeth. Marginal adaptation and fracture resistance are crucial for the long-term survivability and clinical success of such restorations. Virgilite-based lithium disilicate glass-ceramic is a newly introduced material with claims of high strength. However, constructing occlusal veneers from this material of varying thickness has not been investigated.

**Purpose:**

The current study aimed to assess the impact of CAD/CAM occlusal veneer thickness and materials on marginal adaptation and fracture resistance.

**Materials and methods:**

Thirty-two occlusal veneers were constructed and divided into two groups (*n* = 16) based on the CAD/CAM material into Brilliant Crios and CEREC Tessera. Each group was further subdivided into two subgroups (*n* = 8) according to the thickness: 0.6 and 0.9 mm. Occlusal veneers were bonded to epoxy resin dies. The marginal gap was evaluated before and after thermodynamic aging. Fracture resistance and failure mode were evaluated for the same samples after aging. Marginal adaptation was analyzed using the Mann-Whitney U test. Fracture resistance was analyzed using Weibull analysis (α = 0.05).

**Results:**

The marginal gap was significantly increased following thermodynamic aging for tested groups (*P* < 0.001). CEREC Tessera showed a significantly higher marginal gap than Brilliant Crios before and after aging for both thicknesses (*P* < 0.05). CEREC Tessera recorded lower significant fracture load values compared to Brilliant Crios (*P* < 0.05).

**Conclusions:**

Both CEREC Tessera and Brilliant Crios demonstrated clinically accepted marginal gap values. All groups showed fracture resistance values higher than the average masticatory forces in the premolar region except for 0.6 mm CEREC Tessera.

**Clinical implications:**

Reinforced composite occlusal veneers demonstrated more favorable outcomes in terms of marginal gap and fracture resistance at both tested thicknesses compared to virgilite-based lithium disilicate glass-ceramic. Additionally, caution should be exercised during the construction of occlusal veneers from virgilite-based lithium disilicate glass-ceramic with reduced thickness.

## Introduction

Restorative dentistry aims to preserve and maintain the maximum amount of the natural tooth structure. Altogether with the increased esthetic demands by the patients, partial coverage ceramic restorations had been adopted for such purposes [1, 2]. Enamel loss can occur for various reasons, including aging caries and other non-carious lesions such as erosion, abfraction, attrition, and fracture, resulting in breakdown of hard tooth structure, necessitating the construction of subsequent restorations [3].

Dental erosion frequently goes unreported because mineral loss is slow, progressive, and often painless; it is usually discovered at an advanced stage of the condition when there has been a substantial loss of dental tissue [4]. From a biomimetic perspective, such cases should be treated conservatively to prevent unnecessary removal of sound tooth structure, as preservation of tooth structure is the key to sustaining the subtle balance between biological, functional, mechanical, and esthetic considerations [5].

With the advancement of CAD/CAM current materials, occlusal veneers are now utilized as a conservative alternative to traditional overlays or full-coverage crowns for reconstructing the occlusal surface lost tooth structure [6, 7]. Thinner designs, also known as ultrathin occlusal veneers, are now feasible due to the inherent strength and wear characteristics of materials such as lithium disilicate-reinforced glass ceramics and high-performance composite resins [8–10]. Also, it was noticed that patients get motivated when they learn after a treatment planning briefing that minimal or no more tooth structure reduction is required [11].

Advanced glass ceramics, such as lithium disilicate, are stronger than feldspathic porcelain, machinable, etchable by hydrofluoric acid and easily bonded to tooth structure. All the aforementioned advantages expanded their indications to include minimally invasive tooth repairs [12, 13].

Furthermore, the performance of machinable resin composites has increased over the past decade. CAD/CAM resin composites demonstrated increased fatigue resistance compared to ceramics [14, 15].

The mechanical inherited properties of ceramics and resin-based materials differ, raising the question of whether the material can survive in the load-bearing posterior region especially when used in a minimal thickness [16]. Another factor of utmost importance in the success of a restoration is dependent on the marginal adaptation of any performed restoration [17]. Moreover, different marginal preparation designs can affect the mechanical properties and marginal adaptation of occlusal veneers [18, 19].

Disturbance in marginal adaptation facilitates the cement dissolution and leads to subsequent microleakage, development of secondary caries, periodontal disease, pulpal inflammation, and eventual clinical failure of fixed restorations [20, 21].

Occlusal veneers can vary in thickness according to the material used, ranging from 0.3 to 2.0 mm, as reported in the literature for different restorative materials [22, 23]. With that thickness, the challenging events and cyclic loading in the oral cavity are problematic and may lead to cyclic loading fatigue and catastrophic restoration failure [24].

Advanced lithium disilicate (ALD) has recently been introduced into the dental market. It is a glass ceramic composed of lithium aluminum silicate crystals known as virgilite within a zirconia glassy matrix [25]. The long-term clinical survival rate and clinical performance of the newly introduced material in minimal thickness are still lacking. Therefore, the current study aims to evaluate the marginal adaptation and fracture resistance of CAD/CAM advanced lithium disilicate and reinforced composite occlusal veneers of varying thickness restoring bicuspids. The null hypotheses tested were: (1) there would be no significant difference in the marginal gap and fracture load of both tested CAD/CAM materials at any thickness and (2) thermodynamic aging would not influence the marginal adaptation of occlusal veneers.

## Materials and methods

The sample size was calculated using the G power statistical power analysis program (version 3.1.9.4) for sample size determination. A total sample size *n* = 32 (16 in each group) was sufficient to detect a large effect size (d) = 1.55, with an actual power (1-β error) of 0.8 (80%) and a significance level (α error) 0.05 (5%) for two-sided hypothesis test [26].

A total of 32 duplicated epoxy resin dies were divided according to CAD/CAM materials into two equal groups (*n* = 16); Group I, restored with Brilliant Crios (Coltene, Whalendent AG, Switzerland), and Group II, restored with CEREC Tessera (Dentsply/Sirona, USA). The CAD/CAM materials were further subdivided into two groups based on restoration thickness: ultrathin at 0.6 mm and thin at 0.9 mm. The materials used are listed in Table 1.

**Table 1:** List of used materials

Material	Manufacturer	Composition [Lot number]
Brilliant Crios (Ceramic reinforced composite resin)	Coltene, Whaledent AG, Switzerland	Barium glass (Size < 1.0 μm), amorphous silica (SiO_2_, Size < 20 nm), resin matrix (Cross-linked methacrylates), inorganic pigments (ferrous oxide or titanium dioxide) [K34244]
CEREC Tessera (Advanced lithium di-silicate)	Dentsply/Sirona, USA	Lithium disilicate (Li_2_Si_2_O_5_), virgilite 0.5 μm (Li_0.5_Al_0.5_Si_2.5_O_6_), zirconia enriched glass matrix, pigments [16013758]

A typodont tooth (NISSIN, Kyoto, Japan) of an upper first premolar was mounted in an acrylic resin mold using a parallelometer (NEY.TECH, USA) to ensure vertical orientation into the acrylic block. Teeth preparation was performed using a computerized numerical control milling machine (M400 CNC Milling Machine, CENTROID, USA), and a tapered diamond stone with a round end (Intesiv SA) was used. The occlusal surface was anatomically prepared, maintaining the natural inclination of the cusp slopes of 120°. The preparation criteria for both tested groups were occlusal reduction of 0.5 mm and axial wall reduction of 1 mm in length in a cervical direction. The preparation terminated with a deep Chamfer finish line of 0.8 mm. The preparation was finished manually with the aid of an experienced operator using extra fine grit diamond stone and polishing spiral wheels (Sof-Lex, 3 M, USA).

The prepared tooth was duplicated using additional silicone material (Panasil, Kettenbach) to form a 32-epoxy resin (Kemapoxy 103, CMB, Egypt) dies replica.

**Fig. 1:**
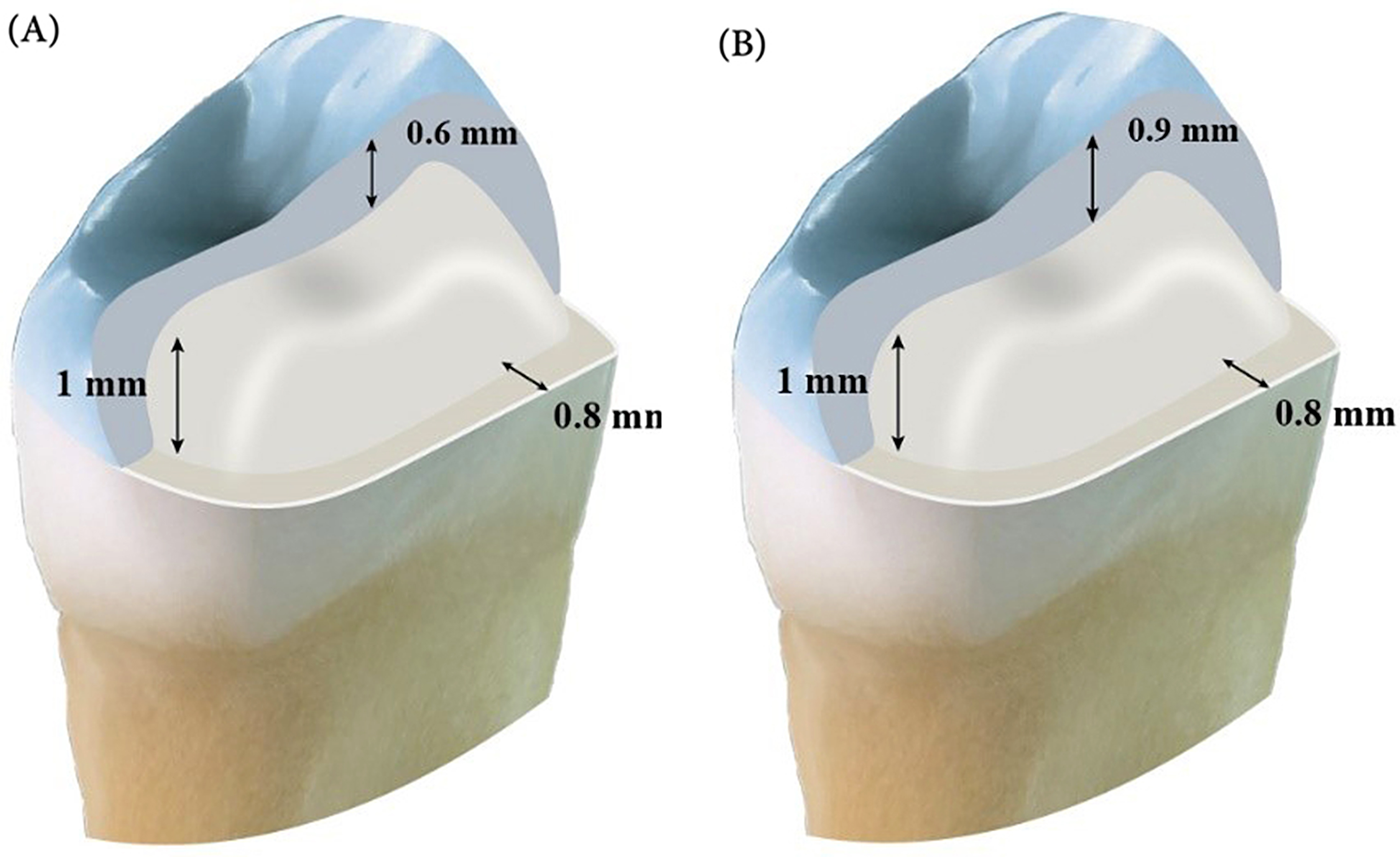
Preparation criteria and occlusal veneer thickness. (**A**) ultrathin occlusal veneers 0.6 mm thickness. (**B**) thin occlusal veneers 0.9 mm thickness

Occlusal veneers were constructed using CAD/CAM technology. A CEREC Primescan intra-oral scanner (Dentsply/Sirona, USA) was used to scan the prepared typodont tooth, and the designing phase was initiated by a highly experienced CAD/CAM designer. For standardization, the restoration parameters were fixed for all the restorations of two tested groups with the radial spacer thickness set at 30 μm [27]. The occlusal thickness of the restorations was 0.6 and 0.9 mm, respectively (Fig. 1), and the milling parameters were consistent for all milled restorations.

CAD/CAM blocks were secured inside the CEREC MC XL milling machine (Dentsply/Sirona). Then, the milling process was initiated using the same design for all restorations, adopting a wet milling protocol for both tested groups. Each restoration was seated and its fit was checked on its corresponding die under magnification of 3× and good lighting conditions before glazing of CEREC Tessera and finishing of Brilliant Crios following the manufacturer’s instructions.

The fitting surface of CEREC Tessera was etched with 9.5% hydrofluoric acid (Bisco, USA) for 20 s, then washed and dried before applying a silane coupling agent (Pentron, USA) for 1 min. For Brilliant Crios occlusal veneers, single bond universal (3M, ESPE, USA) was applied to the fitting surface [28] and thinned with oil-free air (0.2 bar). All the occlusal veneers were bonded to their corresponding dies using conventional dual-cured self-adhesive resin cement (Breeze, Pentron, USA). All samples were stored in distilled water for 24 h before testing.

A four-station multi-model ROBOTA chewing simulator (AD-TECH TECHNOLOGY CO., Germany) integrated with the thermocycling protocol was used to simulate thermo-dynamic aging. Both vertical and horizontal movements were conducted simultaneously in a thermodynamic condition. An 11 kg weight, equivalent to 108 N of chewing force, was applied at a frequency of 1.6 Hz. A total of 250,000 cycles were used for clinical stimulation of a 12-months of service in the oral cavity [29, 30].

The marginal gaps were inspected before and after masticatory simulation using a USB digital microscope with a built-in camera at a magnification of 40× (Microscope, Guangdong, China) for a total of 5 predetermined points on each surface (a total of 20 points for every restoration) and the gap was measured using ImageJ software.

Subsequently, all samples were mounted on a universal testing machine (Intsron Industrial Products, USA) with a load cell of 5kN. Data were recorded using computer software (Bluehill Lite Software, Intsron, USA). A compressive load was applied occlusally using a metallic rod with a round tip (3.6 mm diameter) moving at a crosshead speed of 2 mm/min. In addition, a tin foil sheet was inserted in between to achieve homogenous stress distribution and minimize the transmission of local force peaks. The load was applied until failure and was recorded in Newton.

For failure mode analysis, each sample was photographed using a USB Digital microscope (U500X Capture Digital Microscope, Guangdong) with a built-in camera connected to an IBM-compatible personal computer with a fixed magnification of 10x. Each sample was categorized based on repairability into; mode A: favorable (Repairable) represents the mild failure pattern, veneer failure with intact epoxy resin die, and mode B: non-favorable (irreparable) with failure of occlusal veneer and epoxy resin die [31].

Data was collected and checked for normality using the Kolmogorov-Smirnov test, and homogeneity of variance was assessed using Levene’s test. The marginal gap showed a non-normal distribution, so Mann-Whitney U test was used to compare between tested groups and thickness while Wilcoxon Matched-Pairs Signed-Rank test was used for comparison between before and after cycling. For fracture resistance, Weibull analysis was used for comparison between tested groups (α = 0.05).

## RESULTS

The results showed a significant increase in the marginal gap after masticatory simulation for Brilliant Crios for both 0.6 and 0.9 mm (*P* < 0.001). Similarly, for CEREC Tessera the marginal gap increased after masticatory simulation for both thicknesses (*P* < 0.001). For the comparison between thicknesses, an insignificant difference resulted between 0.6 and 0.9 mm for both Brilliant Crios and CEREC Tessera before and after masticatory simulation (*P* > 0.05). For both thicknesses, CEREC Tessera showed significant higher marginal gap compared to Brilliant Crios before and after masticatory simulation (*P* < 0.05). The marginal gap (µm) before and after masticatory simulation for all tested groups is presented in Table 2.

**Table 2:** Marginal gap (µm) (Mean [95% CI]) comparison of Brilliant Crios and Tessera CAD/CAM blocks with different thicknesses

	0.6 mm			0.9 mm		
	Before	After	p-value	Before	After	p-value
Brilliant Crios	56.7[50.9 to 62.5]	78.3[70.4 to 86.3]	< 0.001	58.4[53.1 to 63.8]	75.4[70.2 to 80.7]	< 0.001
CEREC Tessera	76.6[71.4 to 81.8]	94.6[86.4 to 102.8]	< 0.001	76.8[71.9 to 81.6]	89.8[83.1 to 96.5]	< 0.001
p-value	< 0.001	0.008		< 0.001	< 0.001	

For fracture load, data showed normal distribution and homogeneity of variance were met F(3) = 1.093, *P* = 0.369. For both thicknesses, CEREC Tessera showed lower significant fracture load values than Brilliant Crios (*P* < 0.05). For both materials, 0.6 mm showed lower significant fracture load values compared to 0.9 mm (*P* < 0.05). Values of fracture resistance (N) for all tested groups are presented in Table 3.

**Table 3:** Comparison of fracture resistance between both groups (materials) with different thicknesses

Materials	Thickness	α [95%CI]	β [95%CI]	P10 [95%CI]
Brilliant Crios	0.6 mm	469.6[433.4 to 508.8]^b^	9[5.6 to 22.4]	365.9[303 to 441.8]
	0.9 mm	830.6[774 to 891.4]^d^	10.4[6.7 to 22.6]	668.8[572.6 to 781.1]
CEREC Tessera	0.6 mm	360.4[319.8 to 406.2]^a^	6.1[4 to 13.1]	249.8[192.6 to 323.8]
	0.9 mm	690.2[637.1 to 747.8]^c^	9.1[5.9 to 20.4]	539.3[451 to 644.7]

Different letters within α column indicate significant differences

**Fig. 2:**
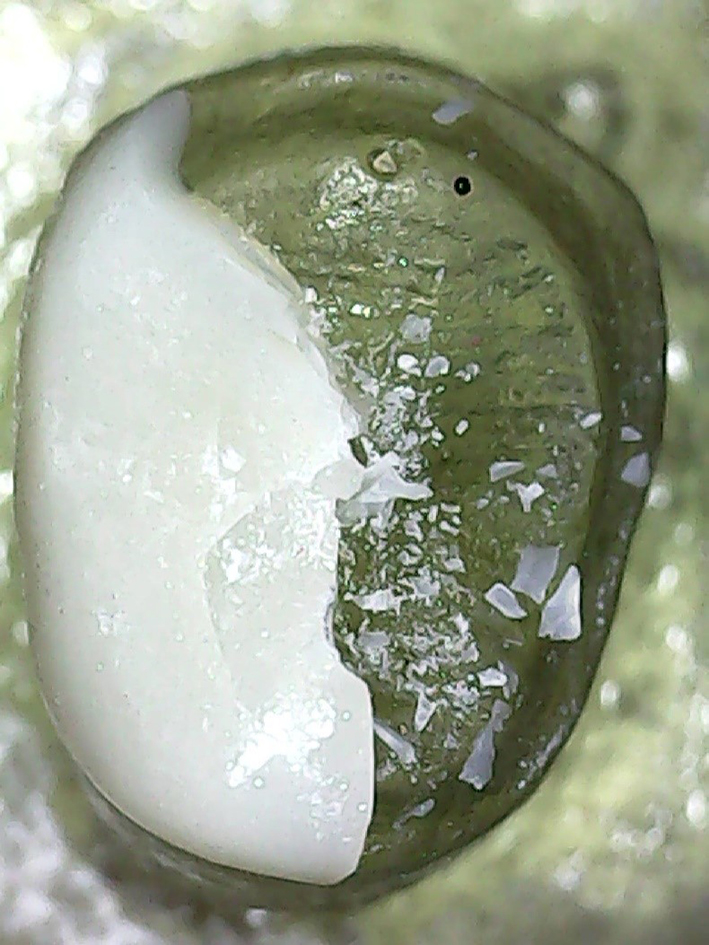
Failure mode A at 10× magnification

All tested samples in all groups showed mode (A): favorable fracture pattern that could be repaired, only fractured restorations. The percentage of mode of failure analysis is presented in Table 4; Fig. 2.

**Table 4:** Failure mode for tested groups

CAD/CAM material	Thickness	Failure Mode	
		A	B
Brilliant Crios	0.6 mm	100.00%	0.00%
	0.9 mm	100.00%	0.00%
CEREC Tessera	0.6 mm	100.00%	0.00%
	0.9 mm	100.00%	0.00%

Mode A: Favorable

Mode B: Unfavorable

## Discussion

In the present study, both null hypotheses were rejected as the results showed a significant difference between the marginal gap and fracture resistance for both tested materials at both thicknesses. In addition, thermodynamic aging influenced the vertical marginal gap.

Minimally invasive procedures in prosthetic dentistry aim to preserve tooth structure without the additional removal of remaining tooth substance. In this study, ultrathin (0.6 mm) and thin (0.9 mm) occlusal veneers were investigated and fabricated from two different CAD/CAM materials. Brilliant Crios is considered a reliable material for treating such cases, while CEREC Tessera is a newly introduced virgilite-based lithium di-silicate with limited evidence of its clinical performance in such a treatment modality.

The results of our study showed that the vertical marginal gap value of CEREC Tessera before and after thermodynamic aging was significantly higher than Brilliant Crios for both tested thicknesses. Furthermore, thermodynamic aging resulted in a significant increase in marginal adaptation for tested groups.

Consistent with our findings, prior research attributed the potential deterioration of marginal integrity in adhesively luted restorations to frequent masticatory forces and thermal expansion variations between the restoration and cement. Thermo-dynamic aging causes thermomechanical stresses on the cement layer, resulting in an increased marginal gap due to its deterioration [32].

Another study compared the marginal adaptation of lithium disilicate and reinforced composite CAD/CAM indirect overlays. They found that reinforced composite had lower marginal gap values compared to conventional lithium disilicate glass ceramics [33]. 

Contrary to the current study, a previous study found that thermomechanical aging reduced the marginal gap. This was justified by the characteristics of the different materials used. The high resiliency of hybrid composite blocks used in the previously mentioned study might have affected the stress transfer, and the material underwent dimensional changes under loading pressure [34].

The results of the current study can be attributed to the higher resiliency of Brilliant Crios occlusal veneers, which have more load absorption and a greater stress-dissipating effect than ceramic Tessera occlusal veneers. This could be supported by a previous in-vitro study, which reported that ceramic overlays had approximately 10% lower marginal adaptation than composite overlays [35].

Notably, all the marginal gaps measured in this study were within the clinically acceptable range of 120 μm [36]. In contrast, other studies reported 160–172 μm is considered clinically acceptable for conventional crowns [37, 38]. The recorded marginal gap values for all tested groups in our study were below the maximum clinically acceptable values in the literature.

In this study, the recorded fracture resistance values revealed that ultrathin occlusal veneers had a lower significant value than thin veneers in this study. Furthermore, CEREC Tessera had lower significant values than Brilliant Crios regardless of thickness. These results agree with a previous study, which found that CAD/CAM thin composite resin occlusal veneers had significantly higher fatigue resistance than ceramic veneers [6, 39].

In the maxillary premolar area, the normal masticatory force is about 450 N, while during clenching, the occlusal force is about 660 N [40]. Our results revealed that all tested groups surpassed the values of normal masticatory force except for ultrathin (0.6 mm) occlusal veneers constructed from virgilite-based glass ceramic. Furthermore, these findings are inconsistent with a previously conducted study that did not recommend the use of lithium disilicate glass ceramics in 0.6 mm thickness in patients with excessive occlusal force in their previous study [6].

A study found that a fracture load of 610 N was observed in fissure areas with a thickness ranging from 0.3 to 0.7 mm. It suggested using lithium disilicate occlusal veneers with a thickness between 0.7 and 1 mm. The variations in findings between this study and others may be due to different research parameters that are not standardized across all studies [41].

Similarly, another study reported that survival was significantly influenced by the restoration thickness when testing lithium disilicate occlusal veneers with different thicknesses (0.5, 0.8, and 1.2 mm). They reported that thicker restorations exhibited a higher survival rate than thinner restorations [42].

Regarding the material effect, Brilliant Crios reinforced composite occlusal veneers showed significantly higher mean fracture loads compared to the CEREC Tessera group with both thicknesses. This finding may be attributed to the synergistic behavior achieved between the polymer matrix of ceramic-reinforced composite, the adhesive system, and resin cement used to have a high composition resemblance. Therefore, they led to superb bonding capacity to the underlying substrate, as indicated by the increased fracture resistance values [43].

Furthermore, our study’s results relatively agree with a previous study that investigated the fracture resistance of occlusal veneers made from three different CAD/CAM materials (e.max, Vita Enamic, and Brilliant Crios). The nanoceramic-reinforced composite demonstrated the highest fracture resistance values [44].

Regarding the mode of failure of the present study, both groups demonstrated that 100% of specimens in both thicknesses showed a repairable mode of failure (Mode A). These findings could be attributed to the low modulus of elasticity of Brilliant Crios reinforced composite occlusal veneers and higher resiliency with more load absorption during loading. Moreover, the presence of polymers in the microstructure of Brilliant Crios reinforced composite made them a more resistant material to crack propagation compared to CEREC Tessera ceramic occlusal veneers. The elastic modulus, similar to that of dentine, is suggested to minimize stress concentration in the restoration and avoid fractures [12, 45].

Failure pattern evaluation showed considerable variation among the previous studies. Two previous studies showed that the most common failure pattern was a cohesive fracture that involved the restoration and cement layer with no damage to the underlying tooth structure, as in the current study [46, 47]. 

It was also reported that fractures or cracks in occlusal veneers were limited to restorative materials [6, 39]. These positive outcomes align with the principles of minimally invasive dentistry. Failures that do not damage the tooth substructure increase the longevity and prognosis of the restored teeth because the veneer can be replaced (repairable failure). Although epoxy dies are suitable as a replacement for natural teeth particularly for mechanical tests as they have similar modulus of elasticity [48] and provide a standardized substrate for testing, confirmatory studies with natural teeth are required. Moreover, different finishline configurations need to be investigated to verify the current finding. Also, Thermo-mechanical aging was simulated for only 12 months, a longer period of simulation still needed to evaluate the long-term success of the occlusal veneers. Clinical research is needed to determine the impact of more complex oral environmental conditions on the mechanical properties of ALD restorations despite the challenges of standardization and variable control.

## Conclusions

Within the limitations of the present study, the following can be concluded:

1. Reinforced composite occlusal veneers showed better marginal adaptation and better tolerance to fracture resistance in both thin (0.9 mm) and ultrathin (0.6 mm) occlusal veneers compared to virgilite-based lithium disilicate.
2. Occlusal veneer with thin (0.9 mm) design provides double the fracture resistance compared to ultrathin (0.6 mm) occlusal veneer which will enhance the survival rate if occlusal veneer for both reinforced composite and virgilite-based lithium disilicate.
3. The use of virgilite-based lithium disilicate in a thickness of 0.6 mm in the premolar region is not advised.

## Data Availability

The dataset used and analyzed data can be available from the corresponding author upon reasonable request.
